# Evaluating the Severity of COVID-19 Infection in Patients With Obstructive Sleep Apnea in Jordan

**DOI:** 10.1155/pm/8077486

**Published:** 2025-11-21

**Authors:** Silvia D. Boyajian, Ensaf Y. Almomani, Muna A. Salameh, Dima Hamarsheh, Sara AlNsour, Riadh Al-Ramadani, Shawkat Al-Tamimi, Husam AlSalamat, Dana Elsalman

**Affiliations:** ^1^Department of Basic Medical Sciences, Faculty of Medicine, Al-Balqa Applied University, Al Salt, Jordan; ^2^Faculty of Medicine, Al-Balqa Applied University, Al Salt, Jordan; ^3^Department of Special Surgery, Faculty of Medicine, Al-Balqa Applied University, Al Salt, Jordan

**Keywords:** COVID-19, hospitalization, obstructive sleep apnea, polysomnography

## Abstract

**Introduction:**

COVID-19 has infected many patients globally, primarily impacting the respiratory system and causing symptoms such as coughing and shortness of breath. Various factors influence the severity of the infection, with obstructive sleep apnea (OSA) being one of them.

**Aims:**

The aim was to investigate the correlation between the severity of OSA and the severity of COVID-19 infection, as indicated by hospitalization, ICU admission, and the duration of recovery from the disease.

**Methodology:**

A retrospective cohort study on OSA patients who follow-up at a tertiary referral hospital sleep clinic and were infected with COVID-19. COVID-19 infection information, such as severity, duration, and vaccination, was collected via phone calls. OSA severity was assessed using the apnea–hypopnea index (AHI). Data were analyzed using SPSS software, and a *p* value < 0.05 was considered significant.

**Results:**

A total of 136 confirmed OSA and COVID-19-positive patients were included in the study. The majority were elderly and obese. Then, 29% of patients had mild, 30% had moderate, and 41% had severe OSA. The severity of OSA was significantly correlated with the COVID-19 type of treatment and recovery duration (*p* value = 0.002 and 0.001, respectively. Severe OSA correlated with higher BMI values. Notably, the type of COVID-19 vaccine, number of doses, and whether the COVID-19 infection occurred before or after vaccination did not affect the severity of OSA.

**Conclusion:**

The severity of OSA and COVID-19 infection were correlated. The management of OSA severity and the control over other comorbidities may lower the chance of severe COVID-19 infection among OSA patients.

## 1. Introduction

COVID-19, an infectious disease brought on by SARS-CoV-2, has so far led to approximately 771 million cases worldwide, including 6.9 million deaths [1]. COVID-19 clinical symptoms are mostly respiratory, including sore throat, cough, and shortness of breath, accompanied by general symptoms like fever, myalgia, and headache. Moreover, extrapulmonary manifestations are reported, such as abdominal pain and diarrhea [2].

COVID-19 symptoms change in severity from asymptomatic to critical disease that may lead to death [2]. The variability in disease severity depends on many factors related to the patient's age, sex, health status, and comorbidities such as hypertension, diabetes, coronary vascular disease (CVD), and liver diseases [3]. Other comorbidities, such as congestive heart failure, chronic renal disease, COPD, and obstructive sleep apnea (OSA), were reported to be associated with severe COVID-19 infection [4].

OSA is a sleep-troubling respiratory disease that is caused by partial or complete recurrent pharyngeal collapses during sleep, leading to multiple episodes of apnea/hypopnea [5]. The prevalence of high-risk OSA is found to be significant among adults (16.8%) [6], adolescents (25%), and children (21%) in Jordan [7]. OSA was initially linked to the severity of COVID-19 in the CORONADO (Coronavirus Sars-Cov2 and Diabetes Outcomes) trial where patients with treated OSA had a higher odds ratio of dying on the seventh day; thus, OSA was thought to be an independent risk factor for poor COVID-19 outcome [8].

Other studies have emphasized the link between OSA and the severity of COVID-19. Research conducted in the United States confirmed that OSA patients had increased mortality rates, identifying the condition as a significant risk factor for mortality in COVID-19 patients [9]. Additionally, poor respiratory outcomes were connected to the severity of OSA, as indicated by a high apnea–hypopnea index (AHI). This was confirmed by the high demand for invasive or non-invasive ventilation. [10]. Also, COVID-19 patients with undiagnosed OSA experienced worse respiratory outcomes, often requiring high-flow nasal oxygen therapy or non-invasive ventilation [11]. Another study showed that patients at high risk for OSA were more likely to require hospitalization or treatment in the ICU [12].

OSA is associated with adverse COVID-19 outcome. OSA causes intermitted hypoxia, endothelial dysfunction and amplification of systemic inflammation all of which increase the risk of severe disease [13] Additionally, the SARS-CoV-2 viral infection and sleep deprivation both enhance the infiltration of neutrophils and monocytes, which exacerbates inflammatory reactions and results in a more severe illness that can lead to sepsis, acute respiratory distress syndrome, or even death [14].

The relationship between the severity of COVID-19 and OSA can be explained by the numerous risk factors that are common to both conditions, including age, diabetes, hypertension, and cardiovascular disorders [14]. Several studies have examined the relation between OSA and COVID-19 outcomes, understanding that comorbidities such as diabetes, hypertension and obesity commonly co-occur among both diseases and may influence the relationships observed. A study reported that OSA was linked to increased risk of hospitalization and developing of respiratory failure among patients with COVID-19 infection even after the adjustment different comorbidities such as BMI, diabetes and hypertension [15]. Additionally, a systematic review and meta-analysis by Hariyanto and Kurniawan found that OSA was significantly linked with higher ICU admission and increased mortality in COVID-19 patients even after adjusting for demographics and comorbidities [16].

This research holds significant value for our community. To the best of our knowledge, this is one of the few studies conducted in the Middle East and the first in Jordan. Thus, our primary goal is to investigate OSA patients with COVID-19 infection in Jordan and determine whether OSA severity, measured by the AHI, is associated with more severe COVID-19 outcomes like hospitalization or ICU admission.

## 2. Methodology

### 2.1. Study Design and Participants

This is a retrospective cohort study that included 310 patients who were diagnosed with OSA by the sleep unit at Jordanian Royal Medical Services, one of the largest medical centers in Jordan, provides treatment to patients from all regions across the kingdom. Patient names and phone numbers were collected from the medical records of patients who visited the sleep unit between September 2020 and September 2023, along with information regarding their OSA diagnosis, severity, and COVID-19 infection. The information about the duration of COVID-19 infection, treatment, and vaccination was collected through phone calls to assess the infection severity. Before collecting information, informed consent was obtained from participants over the phone, ensuring they understood the purpose of the study and voluntarily agreed to participate.

The inclusion criteria involved patients who were 18 years old or more, confirmed their diagnosis with OSA, and they were positive for the COVID-19 infection. Patients who tested negative for COVID-19 by polymerase chain reaction (PCR) test and those with incomplete data were excluded from the study. After applying the inclusion and exclusion criteria, we ended up with 136 OSA patients who were positive for the COVID-19 infection and who agreed to participate in this study. Notably, the COVID-19 subtype was disregarded in patients who tested positive for the virus.

### 2.2. Ethics Statement

This study involves human participants and was approved by the ethical committee of Royal Medical Services and the Institutional Review Board committee at Al Balqa Applied University (Reference number 194/1/3/26). Participants gave informed verbal consent over the phone, ensuring they understood the purpose of the study and voluntarily agreed to participate.

### 2.3. Data Collection

A questionnaire was constructed with three sections. The first section included sociodemographic information (age, BMI, comorbidities, and smoking status). The second section focused on OSA severity, classified based on the AHI into mild (AHI of 5 to < 15), moderate (AHI of 15–30), and severe (AHI of > 30) [17]. AHI was obtained from the patient's medical records through a polysomnography test. To study the severity of COVID-19 infection, the last section included information on vaccination status (vaccine type, number of doses, and its relation to the time of infection) and COVID-19 infection status included the frequency of infections, the type of treatment required, and the time until full recovery, which was determined through patient self-report. Full recovery was defined as the time from the beginning of symptoms until their resolution without the need for additional treatment. Time until full recovery was categorized as follows: less than 1 week, less than 2 weeks, and 2–4 weeks. Consistent with WHO and CDC definitions, recovery taking more than 4 weeks was considered outside the acute COVID-19 stage; therefore, ≥ 4 weeks was used as the upper limit.

Patients were classified according to COVID-19 severity based on the type of treatment received, following WHO therapeutic guidelines. Asymptomatic or presymptomatic patients relied on home remedies only. Patients with mild illness visited the pharmacy and used over-the-counter medication such as paracetamol, aspirin, decongestants, and NSAID. Those who developed moderate illness visited the doctor for medical consultation or the emergency department and underwent chest X-ray evaluation. Finally, patients who progressed severe or critical disease required hospital admission or ICU care.

The first two sections were completed by accessing the medical records of OSA patients. The information for the third section was collected through phone call interviews with patients who agreed to participate in the study.

### 2.4. Statistical Analysis

Statistical analysis was done using IBM SPSS Statistics-Ver 25. Categorical variables are presented as percentages and numbers, while continuous variables are reported as standard deviation and mean. One-sample Chi-square test was used to compare categorical variable and a one-way analysis of variance (ANOVA) test was used to compare continuous variables for three or more groups. *p* value < 0.05 expressed a significant difference.

## 3. Results

### 3.1. The Sociodemographic and Health Information of the Participants

Table 1 presents the sociodemographic and health information of the 136 OSA patients who participated in the study and their correlation with the severity of OSA, measured by the AHI. Among the 136 respondents, 62.5% were males, with a female-to-male ratio of 1:1.6 and a mean age of 53.2 ± 12.9 years. Most of the patients (80.1%) were classified as obese, with a BMI equal to or greater than 30. About 36% of participants were smokers.

The correlation analysis between the sociodemographic information of the patients (age, gender, BMI, and smoking) and the severity of OSA showed no significant correlation (Table 1).

Considering the patients' medical history, 41.9% had diabetes, 75.7% were diagnosed with hypertension, and 39.7% had cardiovascular diseases (CVD). Despite the high prevalence of reported diseases, none showed a significant correlation with the severity of OSA (*p* > 0.05).

### 3.2. COVID-19 Infection and the Vaccination Status of the Participant

The results in Table 2 showed that most of the study participants were vaccinated (91.9%). The majority received the Pfizer vaccine (61.8%), followed by Sinopharm (25%). A large share of the participants had one booster dose (52.2%), and 39.0% had two boosters.

COVID-19 infection was recorded both before and after vaccination, with 63.2% of patients were infected after vaccination. No statistically significant correlation was observed between OSA severity and the vaccination status of patients, number of doses, type of vaccine, and the timing of COVID-19 infection (*p* > 0.05) (Table 2). Noteworthy, the level of OSA severity had no impact on vaccination uptake or infection occurrence before or after vaccination (Table 2).

### 3.3. Association Between the Severity of OSA and the Type of Treatment Needed for COVID-19 Infection

The severity of the COVID-19 infection was evaluated through two approaches. The first approach was according to severity of COVID-19 illness which was classified based on the growth in the necessity of a comprehensive medical intervention such as admitting to the hospital. The second approach was according to the duration of recovery from the COVID-19 infection.

Results in Table 3 showed a significant association between the severity of OSA and the severity of COVID-19 illness (*p* value of 0.002). Asymptomatic/presymptomatic infection and mild illness were more common among mild OSA patients (23.1% and 38.5%), respectively, compared to patients with moderate OSA (2.4% and 29.3%), respectively, and severe OSA patients (5.4% and 17.9%), respectively. In contrast, moderate COVID-19 infection predominated among moderate OSA patients (51.2%) and severe OSA patients (42.9%). Severe to critical COVID-19 infection where patients needed hospital/ICU admissions increased progressively with the severity of OSA, affecting 10.3% of mild OSA patients, 17.1% of moderate OSA group, and 33.9% of severe OSA patients.

Duration needed for treatment was also significantly associated with OSA severity (*p* value of 0.001) as shown in Table 3. The majority of patients with severe OSA (48.2%) required 2–4 weeks to recover from the infection. Then, 56.1% of moderate OSA needed less than 2 weeks for full recovery, and 48.7% of patients with mild OSA recovered within a week.

These results indicate that increasing the severity of OSA is associated with greater COVID-19 severity and longer the duration needed for treatment, suggesting that OSA is linked to adverse COVID-19 outcome.

### 3.4. The Association Between Obesity and the Severity of OSA

**Figure 1:**
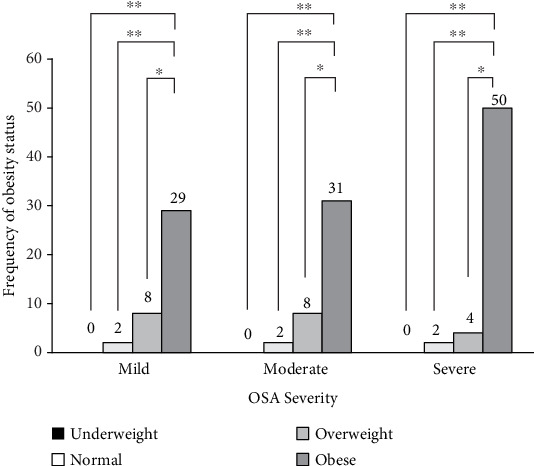
Association between the obesity status and obstructive sleep apnea (OSA) severity using apnea–hypopnea index, *N* = 136. OSA, obstructive sleep apnea; BMI, body mass index in kg/m^2^. *p* values are calculated using two-way ANOVA accounting for multiple comparisons (Tukey). ⁣^∗^*p* value < 0.05 expresses significant differences, while symbols ⁣^∗^*p* ≤ 0.05,^∗∗^*p* ≤ 0.01,^∗∗∗^*p* ≤ 0.001,  and  ⁣^∗∗∗∗^*p* ≤ 0.0001.

When comparing the severity of OSA within the groups based on their BMI classifications, a significant difference was observed among obese patients (BMI ≥ 30) in each group compared to other BMI values. Further, most of the obese patients were among the severe OSA group, indicating that patients with severe OSA had a high BMI (≥ 30), as represented in Figure 1.

## 4. Discussion

The relationship between OSA and COVID-19 severity was evaluated in this study. COVID-19 infection was verified by PCR testing, and OSA patients were clinically identified and categorized by AHI. We approached our findings by focusing on the COVID-19 recovery duration and treatment among the OSA patients. We identified a positive association between the COVID-19 infection and OSA severity. Furthermore, the increase in BMI was associated with an increase in OSA severity.

The relationship between OSA and COVID-19 infection was studied interchangeably. The risk of OSA among COVID-19-infected patients from one side [18, 19] and the COVID-19 infection among OSA patients from other side [20]. Both approaches revealed an association between OSA and the severe forms of COVID-19 infection [18, 19], and severe OSA with COVID-19 infection [20]. In concordance with previous findings, the severity of OSA with the prognosis of COVID-19 infection were correlated.

Herein, about 44% of OSA patients tested positive for COVID-19, in comparison to 18% from a previously conducted study [10]. This difference among OSA patients could be attributed to the genetic epidemiology differences between individuals.

The OSA patients are at a higher risk of developing severe COVID-19 infection compared to normal individuals. The ICU admissions, the need for mechanical ventilation, and the high mortality rate among OSA patients compared to non-OSA individuals support this [15, 21, 22]. The pathophysiology behind this could be attributed to (1) the repeated airway obstructions in OSA cause chronic intermittent hypoxia, which causes oxidative stress, endothelial dysfunction, and upregulates inflammatory cytokines such as interleukin 6 and 8, tumor necrosis factor-alpha, C-reactive proteins, intercellular adhesion molecule-1, vascular cell adhesion molecule 1, causing inflammation amplification [13]. (2) The OSA is also linked to the reduced endothelial nitric oxide activity and microvascular dysfunction, causing impaired vasodilation, tissue hypoperfusion, difficulties in clearing the airways from pathogens, and organ injury when infected [23]. (3) OSA creates a hypercoagulable state through increasing platelet activation, clotting factors, and impaired fibrinolysis, the COVID-19 infection increases the risk for pulmonary embolism and stroke by adding thrombo-inflammation. [22, 24, 25]. (4) OSA activates RAAS (renin–angiotensin–aldosterone system) via hypoxia and sympathetic surges. This leads to fluid retention and eventually hypertension and cardiac strain. Of note, the SARS-CoV-2 virus enters lung cells via angiotensin-converting enzyme 2 (ACE2) receptors, causing imbalance in RAAS and worsen viral effects on lung and cardiovascular system [15, 21]. (5) The continuous positive airway pressure (CPAP) therapy involves the use of a mask to delivers pressurized air to keep the airways open during sleep might increase the risk of spreading respiratory droplets increasing the risk of COVID-19 transmission [23].

The kind of treatment used and the duration of time needed to recover from the infection were considered to determine the severity of the COVID-19 infection. This was primarily demonstrated by the correlation between the severity of OSA and COVID-19 infection [20] and, more precisely, by the higher risk of hospitalization [26], ICU admission, mechanical breathing requirement, and mortality rate [16, 27].

Since the majority of OSA patients were vaccinated for the COVID-19 infection. We discussed the COVID-19 vaccination effect on OSA severity. Noteworthy, neither the COVID-19 vaccination type nor the dosage was influenced by the OSA severity. Further, the COVID-19 infection before and after vaccination showed no significant difference in OSA severity. Our findings are in line with the previously published data, which found no correlation between COVID-19 vaccination and the severity of OSA among older adults [28]. Indicating that OSA severity does not impair vaccine-induced antibody response.

We also evaluated the comorbidities effect on both OSA disease and COVID-19 infection. As previously reported, comorbidities weaken the immune system and participate in increasing the chance of COVID-19 infection [29]. Herein, diabetes, hypertension, and cardiovascular diseases were prevalent among OSA patients (> 50%). Comorbidities, however, did not substantially correlate with the severity of OSA; this may be because OSA patients manage their comorbidities adequately [29].

Consistent with previous findings, obesity is highly prevalent among OSA patients as well as the COVID-19-infected individuals [30, 31]. The OSA is strongly linked with obesity, insulin resistance, and metabolic syndrome [30]. This could be attributed to fat deposits in the patients' upper respiratory tract that narrow the airways and restricts airflow and oxygen supply into the body, in addition to the hypoxia and apneic episodes that happen consequently [31].

Our study's strength stems from the fact that it not only confirms previous findings about the association between OSA and COVID-19 infection but also shows a correlation between the severity of OSA and the severity of the COVID-19 infection through evaluating the followed treatment approach and recovery duration from the infection.

However, this study is limited because it relies on data collected from the hospital existing records, which may be incomplete, inaccurate, or missing information such as the patient's history of pulmonary diseases, the total sleep times (TSTs), patients who passed away, information about the COVID-19 subtypes, and the OSA treatment, which may have provided insight into the relationship between COVID-19 treatment and mild OSA. Furthermore, the small sample size may cause selection bias that influenced the generalizability of the study findings.

## 5. Conclusion

To sum up, OSA is one of the concomitant disorders that exacerbate the severity of COVID-19 infection. The severity of OSA is correlated with the severity of COVID-19 infection. Severe OSA patients needed a longer time to recover from the COVID-19 infection and were more likely to need to be admitted to the hospital or intensive care unit. It is imperative to recognize that patients with OSA are high-risk individuals who require extra safety precautions when managing their illness. Furthermore, managing the severity of OSA and other associated comorbidities is essential to lowering the risk of COVID-19 infection severity in OSA patients.

## Figures and Tables

**Table 1:** The sociodemographic and health information of the study participants and the correlation to obstructive sleep apnea severity using the apnea-hypopnea index, *N* = 136.

**Variable**	**N** ** (%)**	**OSA severity** **Chi-square (*p* value)**
Gender, *N* (%)		0.754
Male	85 (62.5)	0.298
Female	51 (37.5)	0.390
Age in years, mean ± SD	53.2 ± 12.9	
Male	49.6 ± 13.4	
Female	59.2 ± 9.5	
BMI in kg/m^2^, mean ± SD	35.7 ± 7.1	
Male	38.9 ± 7.3	
Female	33.8 ± 6.3	
Obesity, *N* (%)		0.340
Underweight	0 (0.0)	
Normal weight	6 (4.4)	
Overweight	20 (14.7)	
Obese	110 (80.1)	
Smoking, *N* (%)	43 (31.6)	0.436
Medical history, *N* (%)		
Diabetes	57 (41.9)	0.246
Hypertension	87 (75.7)	0.936
Cardiovascular diseases (CVDs)	54 (39.7)	0.897

*Note:* The *p* value represents Pearson Chi-square.

Abbreviations: AHI, apnea–hypopnea index, BMI, body mass index; CVD, coronary vascular disease; *N*, number; OSA, obstructive sleep apnea; SD, standard deviation.

**Table 2:** The COVID-19 infection and the vaccination status of the OSA patients, sample *N* = 136.

**Status**	**N** ** (%)**	**OSA severity** **Chi-square (** **p** ** value)**
Vaccinated, *N* (%)	125 (91.9)	0.212
Type of vaccine, *N* (%)		0.419
Pfizer	84 (61.8)	0.215
Sinopharm	34 (25.0)	0.439
AstraZeneca	11 (8.1)	0.307
Sputnik-19	2 (1.5)	1.000
How many doses of the vaccine, *N* (%)		0.541
One	1 (0.7)	NA
Two	71 (52.2)	0.594
Three	53 (39.0)	0.397
COVID-19 infection relevant to vaccination		0.515
Before taking the vaccine	46 (36.8)	0.915
After taking the vaccine	79 (63.2)	0.205

*Note: p* values are calculated using one-sample Chi-square test.

Abbreviation: OSA, obstructive sleep apnea.

**Table 3:** The severity of COVID-19 infection of study participants and correlation to obstructive sleep apnea severity using the apnea-hypopnea index, *N* = 136.

	**Severity of OSA**				
	**Mild (** **n** = 39**)**	**Moderate (** **N** = 41**)**	**Severe (** **N** = 56**)**	**Total ** **N** ** (%)**	**Chi square ** **p** ** value**
Severity of COVID-19 infection based on type of treatment required					0.002⁣^∗^
Asymptomatic/presymptomatic (home remedies only)	9 (23.1)	1 (2.4)	3 (5.4)	13 (9.6)	
Mild (using over-the-counter medication)	15 (38.5)	12 (29.3)	10 (17.9)	37 (27.2)	
Moderate (visiting doctor or ER)	11 (28.2)	21 (51.2)	24 (42.9)	56 (41.2)	
Severe-critical (hospital or ICU admission)	4 (10.3)	7 (17.1)	19 (33.9)	30 (22)	
Duration needed for treatment					< 0.001⁣^∗^
Less than 1 week	19 (48.7)	6 (14.6)	8 (14.3)	33 (24.3)	
Less than 2 weeks	14 (35.9)	23 (56.1)	21 (37.5)	58 (42.6)	
two to 4 weeks	6 (15.4)	12 (29.3)	27 (48.2)	45 (33.1)	

*Note:* Models are adjusted for demographic, vaccination, lifestyle, and comorbidity covariates (gender, age, BMI, smoking, vaccination type, diabetes, hypertension, obesity, and cardiovascular problems). *p* values are calculated using Pearson Chi-square.

Abbreviations: NSAID, nonsteroidal anti-inflammatory drugs; OSA, obstructive sleep apnea.

⁣^∗^*p* value < 0.05 expresses significant differences.

## Data Availability

The data that support the findings of this study are available from the corresponding author, upon reasonable request.
